# Energy-Efficient Integration of Continuous Context Sensing and Prediction into Smartwatches

**DOI:** 10.3390/s150922616

**Published:** 2015-09-08

**Authors:** Reza Rawassizadeh, Martin Tomitsch, Manouchehr Nourizadeh, Elaheh Momeni, Aaron Peery, Liudmila Ulanova, Michael Pazzani

**Affiliations:** 1 Department of Computer Science and Engineering, University of California Riverside, Riverside, CA 92521, USA; E-Mails: apeer001@cs.ucr.edu (A.P.); Lulan001@ucr.edu (L.U.); michael.pazzani@ucr.edu (M.P.); 2 Design Lab, The University of Sydney, Sydney 2006 NSW, Australia; E-Mail: martin.tomitsch@sydney.edu.au; 3 Vienna University of Technology, Vienna 1040, Austria; E-Mail: nourizadehbarabi.m.at@ieee.org; 4 Multimedia Information System Group, University of Vienna, Vienna 1090, Austria; E-Mail: elaheh.momeni.roochi@univie.ac.at

**Keywords:** wearable, smartwatch, mobile sensing, prediction, energy efficiency, lifelogging, quantified self

## Abstract

As the availability and use of wearables increases, they are becoming a promising platform for context sensing and context analysis. Smartwatches are a particularly interesting platform for this purpose, as they offer salient advantages, such as their proximity to the human body. However, they also have limitations associated with their small form factor, such as processing power and battery life, which makes it difficult to simply transfer smartphone-based context sensing and prediction models to smartwatches. In this paper, we introduce an energy-efficient, generic, integrated framework for continuous context sensing and prediction on smartwatches. Our work extends previous approaches for context sensing and prediction on wrist-mounted wearables that perform predictive analytics outside the device. We offer a generic sensing module and a novel energy-efficient, on-device prediction module that is based on a semantic abstraction approach to convert sensor data into meaningful information objects, similar to human perception of a behavior. Through six evaluations, we analyze the energy efficiency of our framework modules, identify the optimal file structure for data access and demonstrate an increase in accuracy of prediction through our semantic abstraction method. The proposed framework is hardware independent and can serve as a reference model for implementing context sensing and prediction on small wearable devices beyond smartwatches, such as body-mounted cameras.

## 1. Introduction

The advent of wearable devices, such as fitness trackers and smartwatches, provides new opportunities for quantifying human behavior to an extent, which was previously not possible [1]. The rich sensor interfaces of wearable devices, in addition to their computing and networking capabilities, make them a promising platform for continuously monitoring people’s behaviors through collecting contextual data. Contextual data collected through small devices, such as wearables and smartphones, not only allows people to retrospectively reflect on their daily behavior, which may trigger behavior change, but can also be used to predict aspects of human behavior [2], e.g., speech therapy [3]. There are several applications that use the context sensing capabilities of smartphones for prediction, such as energy-efficient location sensing [4], personalization of the user interface [5] and trip planning [6]. In particular, context sensing has seen considerable attention, and there are a number of frameworks available to developers to integrate context sensing into their applications, such as Intel’s Context Sensing SDK (https://software.intel.com/intel-context-sensing-sdk).

Despite the rich sensors that mobile and wearable devices offer, there are a number of challenges that limit the application of context sensing features in real-world applications [7]. These challenges can be listed as follows: First, the small capacity of batteries in mobile and wearable devices limits the use of resource-consuming sensors, such as GPS, which can lead to sparsity in the collected data [8]. Second, the small scale of sensors results in less accurate sensor data for sensors using radio frequency, which includes GPS, Bluetooth and WiFi [9]. Third, devices are not always in close proximity to the user, which restricts continuous context sensing, especially for smartphones that are not carried by the user at all times [10]. Fourth, contextual data that are automatically recorded are highly sensitive, and privacy protection of the data is still an ongoing topic in context sensing research [11,12]. Finally, sensors can be manually disabled through user intervention, again causing the risk of sparsity and uncertainty in the collected data.

These challenges will be exacerbated when we apply data analysis techniques on contextual data. Nevertheless, there are some promising approaches that have been proposed for mining data collected through smartphone devices, including Mobile Weka [13] and MobileMiner [14]. However, due to the limitations discussed above, there is not much research on data analysis techniques for mobile and wearable data streams compared to other fields, such as social media, financial systems and image recognition [15].

Furthermore, data analysis algorithms, such as data mining or prediction, are usually processor intensive and therefore performed in the cloud [16,17], which makes the target application network dependent. Most of the existing smartwatches use a paired smartphone as a network medium to connect to a cloud. Therefore, a connection between a smartwatch and the cloud requires a Bluetooth connection between the smartwatch and the smartphone. Due to device proximity and the resource limitations of smartwatches, establishing such a connection frequently and constantly is expensive and not practical (see Section 5.3.1). Therefore, we believe a real working system requires shifting the data analysis process from the cloud to the device. To our knowledge, there are no frameworks to assist with the integration of data mining (or prediction in our scenario) on the small wearable devices.

To bridge this gap, we introduce a novel generic continuous context sensing and resource-efficient prediction framework for small and wearable devices. The integration of prediction in context sensing tools is an emerging paradigm in mobile and ubiquitous computing, which was coined anticipatory sensing by Pejovic and Musolesi [18]. We demonstrate our approach through an implementation for wrist-worn devices, *i.e.*, smartwatches. We chose smartwatches as a platform over other types of wearables, as they are becoming more widely used and less prone to being lost or forgotten [9]. Smartwatches are smaller in scale than smartphones and, thus, pose more limitations than smartphones.

Such a framework requires addressing the limitations discussed above, especially battery constraints. Furthermore, to ensure its wide applicability, it needs to be generic (application-agnostic) and integrated. Its prediction and sensing module should further be independent from the underlying device-specific sensors to ensure that it is flexible and can be used on models from different manufacturers and by different types of wearable applications. According to Rehman *et al*. [15], there are three classes of information sources: device-specific, operating system-specific and user-specific information. Our work focuses on user-specific information, although the distinction is somewhat blurred for some aspects. For example, battery performance could be considered both device-specific, as well as user-specific information, since it is valuable for the user to have an awareness and overview of this type of information.

Although there are several promising context-sensing and predicting tools for smartphones (e.g., [13,14,16,19]), to our knowledge, this is the first framework that integrates context sensing and prediction, while being specifically designed for smartwatches (due to its overall resource efficiency and on-device prediction). There are use case-specific prediction embedded medical devices [20], but they are not generic.

We provide an implementation based on this framework in a quantified self-based lifelogging app for smartwatches, “insight”. Please note at this time, the “insight” code is open source and available here: https://github.com/rezar/insight. Due to the use of third party libraries and their licenses, we might have to remove the public access in the future. However, interested readers can send us an email to receive a custom-built version of insight without third party libraries.

All of our continuous context sensing components (except the ambient light sensing component) are inspired by current approaches for mobile sensing, but adapted for smartwatches. Our novel prediction module is significantly different from existing prediction modules, since: (i) it is light and energy efficient, so that frequent execution of this module has only a minor impact on battery utilization; (ii) it operates on-device and removes the need for data transfer, which is a highly network- and battery-consuming process; (iii) its algorithm is sensor independent, which means that it can operate with any timestamped information object, regardless of the sensor type, making our approach flexible to handle different timestamped contextual data; and (iv) it benefits from a novel semantic abstraction that has been applied to both the temporal aspect and content of the contextual data.

Although we recommend keeping the process on the device, it should be pointed out that computationally-complex processes should be handed over to a device with more advanced computing capabilities. There are efforts to optimize computationally-complex applications, such as web search [21], for small devices. Nevertheless, it is not cost effective to execute algorithms with high computational complexity on small devices due to the resource usage of these algorithms. Therefore, we recommend substituting those computationally-complex algorithms with lighter versions, such as the one we describe in Section 4.2.3.

The remainder of this paper is organized as follow: First, we describe related work. Next, we list notations for the design and implementation of our proposed system. Then, we describe the architecture of our framework. This is followed by an evaluation of our implementation through a deployed real-world app, “insight”. Finally, we conclude the paper and present directions for future work.

## 2. Related Work

Our research contributes to the fields of context sensing and context prediction. Therefore, we describe three relevant categories of related work. The first category focuses on continuous context sensing frameworks. The second category focuses on the prediction and integration of context sensing and the prediction system on mobile devices. The third category focuses on previous works that proposed context sensing and reasoning applications for smartwatches or other wrist-mounted wearables.

### 2.1. Continuous Context Sensing

There are several context sensing approaches that are focused on either localization and indoor location detection or activity recognition. Song *et al*. [22] provide a survey of several works that try to benefit from a combination of different sensors to support indoor localization, such as camera, sound, WiFi, RFID, Bluetooth, or even single sensors, such as microphone (ultrasonic chirps of mobile devices) [23].

Several approaches benefit from sensor fusion to extract location, such as WiFi and microphone [24] or GPS and accelerometer [25]. Lara *et al*. [26] provide a survey of activity recognition approaches, which again includes single-sensor [27], as well as sensor fusion approaches [25], such as accelerometer and WiFi.

A number of approaches have been proposed that provide general context sensing, which is not trimmed for a specific use case. This development is likely driven by the fact that continuous context sensing is maturing, therefore moving from application-specific approaches to more holistic and flexible approaches. Early examples of context sensing efforts are focused on data collection, such as the Context Toolkit [28], Sensay [29] and Context Phone [30]. However, there is an increasing number of context sensing approaches designed for smartphones, which primarily focus on energy efficiency while collecting contextual data [16,19,31,32]. For instance, these approaches try to preserve battery life by increasing the sampling rate when a user is moving (detected by an accelerometer sensor) and reduced sampling while the user is stationary [33,34]. Another approach is to analyze each sensor’s effect on energy consumption and to use low energy-consuming sensors/policies in combination with short infrequent bursts of high accuracy and high-energy sensors/policies [31]. Although our approach is different, it benefits from previous work by incorporating their findings into the design of our framework. For instance, we took inspiration for implementing sensor independency while collecting data from the UbiqLog framework [19].

Energy efficiency remains a major issue, but continuous context sensing is maturing, which has led to the release of commercial libraries, such as Intel’s Context Sensing SDK.

### 2.2. Context Prediction and Reasoning

Several studies have focused on analyzing raw contextual data, mostly originating from smartphones for prediction and reasoning. Bettini *et al*. [35] provide a survey of context-based reasoning and list reasoning approaches that use ontology for reasoning. Ma *et al*. [17] propose a spatio-temporal generalization (*i.e.*, 18:00–8:00 to home, 8:00–18:00 to work) and substitute the genre of the application instead of their names to normalize and resolve the context sparsity. They subsequently use a constraint-based Bayesian matrix factorization to extract similarities among users’ behavioral patterns. Their approach is very promising, but the location estimation based on two temporal aspects of the day could neglect the details of human behavior because of such a generalization. ACE [16] and MobileMiner [14] frameworks use association rule mining to predict contextual activities that are occurring together, which have applications in energy-efficient sensing. They do not provide a generalization on context data and rely on original data. Lifestreams [36] is another context data collection approach that collects context information and manual user’s information. Lifestreams proposed a change point detection algorithm for context data and, based on the survey result, tries to identify correlations between human activity and their feedback. Its change point detection algorithm requires 30 days as a cold start to identify change points.

Our approach is holistic, similar to ACE [16] and MobileMiner [14]. In contrast to these works, we do not use association rule mining for the prediction. Our algorithms compute the inferences based on the time of the day (similar to human perception of time), which can also function in single-sensor settings and does not require more than one sensor datum being available. Moreover, our approach has the advantage of being sensor independent and can operate on a very short cold start state, only requiring a minimum operation of two days.

### 2.3. Integrated Frameworks for Wrist-Worn Wearables

eWatch [24] provides a location recognition based on a combination of light and audio sensors. It relies on learning algorithms based on the nearest neighbor principle and uses 1-NN classification for online classification in real time in order to estimate indoor location. In another work [33], the authors used eWatch as a base platform and developed a wrist-worn wearable for activity recognition. They used manual labels to evaluate the accuracy of their approach. They concluded that selective sampling decreased energy consumption while maintaining high accuracy. Berlin and Van Laerhoven [37] presented a wrist-worn device to detect human activities with the aim to model mood and physical activity for psychiatric patients. They use motif discovery to characterize the routine activities that users perform. Activity recognition is a well-known use case for smartwatches and wrist-worn wearables. Still, there are efforts to create more energy-efficient data collection approaches. A recent study by Berlin *et al*. [34] analyzes the energy efficiency characteristics of sensing components. In particular, they analyze the energy impact of different SD card brands, sample controller components and OLED displays. Dong *et al*. [38] use a smartwatch to measure meal intake. They use a gyroscope mounted sensor worn on the users’ wrist and a novel algorithm to detect eating through hand, spoon, chopstick, *etc*. Chowdhury *et al*. [39] describe an approach that uses a similar algorithm for gesture recognition.

All of the works described above rely on a combination of hardware and software solutions that are designed for a specific use case. This limits the adaption of the underlying algorithms for other use cases. Our approach considers the varying sensors and programming capabilities of smartwatch models. Furthermore, our approach is generic enough to be used for different use case scenarios, instead of being trimmed for a specific goal. To demonstrate its generic feature, we have implemented prediction for both physical activity and the battery.

## 3. Notations and Definitions

In this section, we define a number of terms and their interpretation in the context of the proposed context sensing and prediction framework.

*Activity:* We define *Activity* as a fine-grained unit of contextual data. All activities that a context sensing application can collect are timestamped. Moreover, since a context sensing system is multimodal, there are different sources of information. An activity information object is being used as the basis for sensing and prediction. Activities are being sensed and recorded by the device, and each activity includes a three-tuple of a timestamp, source name and data, presented as <T,S,D,>. *S* is the source name and describes the sensor or data source associated with the data, e.g., Bluetooth, physical activity, notification. *D* captures the value associated with this source and presents the data object. The data object can contain more than one element. In our implementation, we store activities in JSON format. *T* is the timestamp. The timestamp can be discrete, such as the time a notification arrived, e.g., <12:02:01 15.Jan.2015, "application", "gmail">, or continuous, such as a timestamps for a sequence of steps a user takes while walking, e.g., <[12:27:12-12:41:53] 15.Jan.2015, "activity", "walk">.

*Generalized Activity:* In order to be able to provide a higher level abstraction of activities that can be used by the prediction algorithm, a generalization of the activity data object, *D*, and its timestamp, *T*, are required. Therefore, a generalized activity will be presented as $<T^{\prime },S,D^{\prime }>$. The source name is not generalized, but timestamp and source data are converted to $T^{\prime }$ and $D^{\prime }$ through our semantic abstraction. For instance, the two previous activity examples will be generalized as: <12:30 15.Jan.2015, "application", "communication"> and <12:00-13:00 15.Jan.2015, "activity", "walk 345 steps">

*Profile:* We define *Profile* as a set of *n* generalized activities *a* that were repeated among the weekdays $d_{x}$ and with the minimum number of repeats *θ*. For instance, if we have running, from 18:00–19:00, and this activity happened on Tuesday and Thursday, the profile of the user contains a record for these two similar activities as: {"activity":"running", "time":"18:00–19:00", "days"="{"Tuesday", "Thursday"}", "*θ*":2} . Equation (1) shows the notation for the profile with the number of generalized activities *n*:

(1) $$profile=\left\{\begin{array}{c} \overset{n}{\underset{i=1}{⋃}}\left(a_{i},d_{x},\theta \right)\\ d_{x}\subseteq weekdays \end{array}\right.$$

If an activity does not repeat itself during the comparison, this activity is not frequent, and thus, it will not be considered in the profile object. Therefore the minimum for *θ* is two. Connecting the profile to a notification component constitutes the prediction features of our approach.

Since this model handles all information as a three-tuple of <S,D,T>, it is independent from its underlying sensor, and *S* could be any information source. For instance, such a model can be used for body-mounted cameras (which can annotate the location of pictures) to create a user profile, and a profile could be as follows: {"activity":"gym", "time":"17:00-19:00", "days"="{"monday", "wednesday", "friday"}", "*θ*":3}.

## 4. System Design

As stated earlier, to our knowledge, there is currently no generic context sensing framework for smartwatches or other wearable devices. Previously, the lack of generic tools originated from different hardware configurations of devices [31]. New smartwatch operating systems, such as Pebble (https://getpebble.com), Android Wear (www.android.com/wear) and Apple Watch (https://www.apple.com/watch), provide SDKs that could bridge this gap. The architecture we propose here and the proposed data model are both flexible enough to allow developers to implement them on different platforms or even different wearable devices.

**Figure 1:**
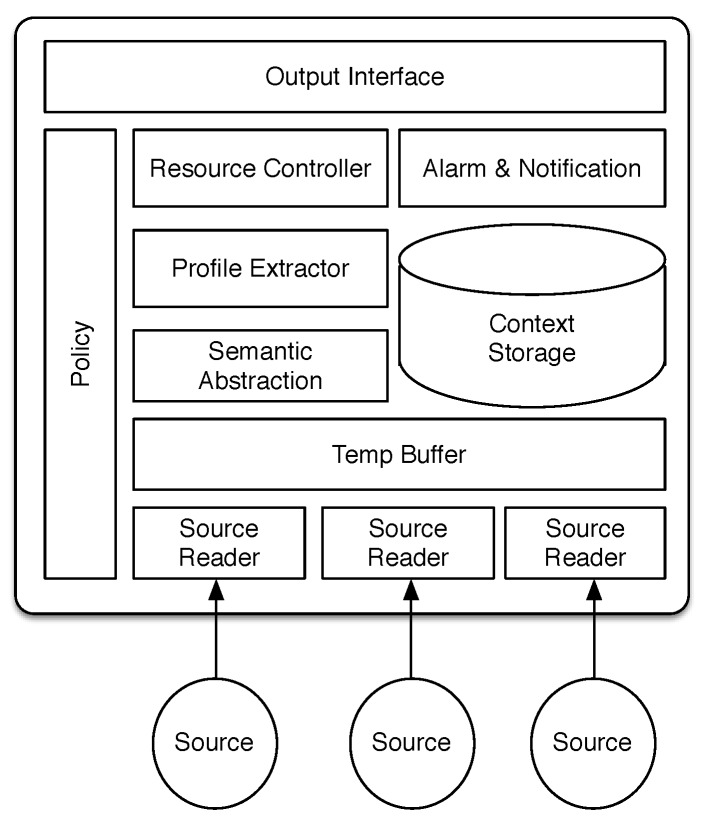
Components of the proposed context sensing and prediction framework.

In this section, we first describe our context sensing approach and its components in the architecture (Figure 1). We then describe our prediction algorithm and its related components. We categorize the features of each component below its description.

### 4.1. Sensing and Data Collection

Opportunistic sensing [40] is the process of collecting, storing and processing data related to everyday human activities. Resource efficiency is a major challenge in opportunistic sensing [15]. This is due to the fact that ideally, the sensing process should run in the background of the device (which has limited resources) and collect information continuously at all times (24/7). Continuous sensing could be done either as interval based or event based. Interval based means fetching the data from a sensor based on a specific time interval, and event based means read the sensor data when a specific change has occurred. Since an event-based strategy is more conservative and, thus, more energy efficient, our implementation favors event based over interval based. However, some source data will be read as interval based.

Table 1 summarizes the data fetching policies that were used in the implementation of our framework. It is worth pointing out that our implementation tries not to use raw sensor data and instead focuses on rich and human-readable sensor information. For instance, it does not collect accelerometer data, but instead, it collects human activities from Google Fit, such as running, walking, *etc*. (similar to examples in Section 3). For sensor data that is not human readable, such as the data from the ambient light sensor, the data are converted to a semantically-rich information object. The semantic conversion strategy will be explained later in this section. The prediction model uses semantically-enriched information, and thus, we remove the performance burden of storing raw data (which is larger than annotated data).

**Table 1:** Implemented sensors and their sensor data fetch policies.

Sensor Name	Fetch Style	Read and Record Policy
Activity	Event and interval	Once a day or when the user opens its UI
Heart rate	Event and interval	Once a day or when the user opens its UI
App usage	Interval	Immediately when the listener provides data
Battery	Event and interval	Interval based on 5% change events
Notification	Event	As appears
Bluetooth	Event	As connection status changes
Ambient light	Interval	Customized interval

With the exception of the ambient light sensor, other source reading policies are based on previous works [15,19]. Continuously sensing ambient light is not resource intensive [31], but frequently writing to the disk is resource intensive (see Section 5.2.1). Ambient light is further changing at a very fast rate, and therefore, event-based collection is not ideal. To resolve this issue, we provide a novel approach to record ambient light based on real individual state changes, which has been identified through our formative experiments.

**Figure 2:**
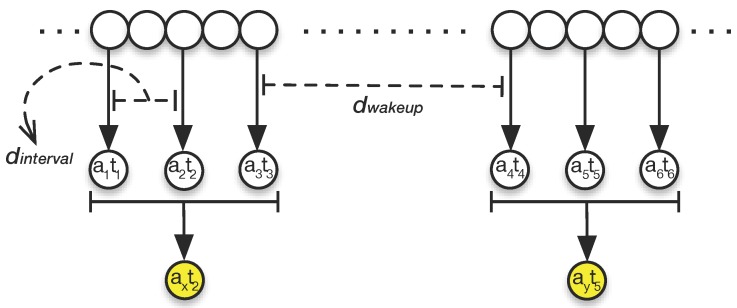
Ambient light calculation in three stages. Each circle represents a data point. Every $d_{wakeup}=10$ min, our system samples three data points with $d_{interval}=30$ s between each sampling, *i.e.*, $a_{1}t_{1},a_{2}t_{2},a_{3}t_{3}$ and $a_{4}t_{4},a_{5}t_{5},a_{6}t_{6}$. The average of the three points is stored with the timestamp of the middle point, *i.e.*, $a_{x}t_{2},a_{y}t_{5}$.

The *Sensor Reader* samples the ambient light three times every $d_{wakeup}$ minutes. The Sensor Reader waits for $d_{interval}$ seconds between each sampling. Then, the average of these three samples is recorded in the dataset along with the timestamp of the second reading. It is not possible to suggest optimal values for $d_{wakeup}$ and $d_{interval}$, as their configuration is application specific. However, in our implementation “insight”, we set these values to 10 min for $d_{wakeup}$ and 30 s for $d_{interval}$. Figure 2 shows the sampling policy for ambient light in our implementation.

#### 4.1.1. Decoupled Sensing Modules

We decouple the context sensing component from other components of the application in order to maintain the flexibility and multimodality of the sensing platform. This makes the framework extendable to accept different types of sources and, thus, makes it flexible to be used for different use case scenarios. In order to provide this feature, each source (sensor) is associated with its own *Sensor Reader* (Figure 1). When a source fails to provide data, only its Sensor Reader raises an error (other Sensor Readers will not get blocked); this therefore does not harm the operation of other sources.

This design is based on the “black board pattern” [19]. The *Policy* component in Figure 1 is a data dictionary that hosts source configuration and application settings, such as data retention period, sensing policies and configurations that have are described in Table 1. The *Resource Controller* component is responsible for starting and stopping Sensor Readers based on their associated configurations in the *Policy*. In the implemented prototype, the Policycomponent is distributed across a database (for sensor settings) and a file (for battery-aware sensing, data retention policies, *etc*).

#### 4.1.2. Energy-Efficient Sensing

Energy efficiency is recognized as one of the most important challenges for mobile and wearable devices [18,41,42,43]. Battery capacity drops cubically with the size of the battery [41]. Several works target the issue of energy efficiency for sensing and collecting contextual information [16,19], highlighting the importance of energy efficiency for continuous context sensing. Zhuang *et al*. [44] and Pejovic-Musolesi [18] both provide an overview of energy-efficient methods for continuous context sensing.

Zhuang *et al*. [44] describe four approaches being used to control resource efficiency and to preserve battery: (i) energy profiling, which satisfies quality of service and quality of experience, e.g., adapting sensing accuracy to the battery state; (ii) substituting resource-consuming sensors with lower resource consuming sensors, e.g., substituting GPS with WiFi; (iii) power saving design based on leveraging history and caching; and (iv) suppressing sensing based on the context, e.g., when a device is in an indoor environment, GPS sensing is not necessary. From the perspective of sensing, Pejovic and Musolesi [18] describe two types of energy-efficient sensing: adaptive sampling, which changes the sampling frequency based on energy level, and preferring low-power sensors over higher-power ones.

We use the first method of Zhuang *et al*.’s taxonomy. We further use the first method of Pejovic and Musolesi’s taxonomy. Through the Policy component, we allow developers to define a “sensing suspension policy” per sensor based on battery state.

The following shows an example of the records inside the Policy file for this approach. If the battery capacity goes below 15%, then the system should stop sampling ambient light data:

{"sensing":{"batterylevel":{15},
 {"sensor":{"light"},"action":{"stop"}}}}


Moreover, similar to the accelerometer, the ambient light sensor samples the data very rapidly, and continuously writing ambient light data onto the disk is not energy efficient. Our framework samples and stores ambient light data efficiently from both the energy and storage perspective. Section 5 demonstrates the energy efficiency and disk impact of our ambient light sampling in detail. This section provides a more detailed discussion of the energy efficiency aspects of the proposed framework.

#### 4.1.3. Data Storage

After source data have been collected, they will be sent to *Temp Buffer*. This buffer also acts as a temporary storage for sensor data to avoid synchronous writing access to files, which would affect the system performance. When the number of records inside the Temporary Bufferreaches a specific threshold, which is configured in the Policy component, the system writes the buffer content into the *Context Storage*.

The following shows a record inside the Policy file if the Temp Buffer size reaches the threshold (e.g., 20 entries), the system should flush all of the buffer data into the storage.

{"sensing":{"buffersize":{20},
 {"sensor":{"all"},"action":{"write"}}}}


The Context Storageis a database that holds data using a folder structure. The folder structure includes a folder for each sensor. Inside each folder, a file is created per day that hosts the context sensing logs. If the user intends to open a UI that includes sensor data in the buffer, all related data in the buffer will be flushed to the storage. In the evaluation section, we analyze the efficiency of this folder structure compared to other Context Storage approaches.

#### 4.1.4. External Service Calls

We have described that our framework could operate independently as a standalone module on a device. However, our implementation is based on Android Wear, and at the time of writing this paper, there are some limitations regarding the access of information directly from the smartwatch. Android Wear requires an Android smartphone for full functionality. This is due to the fact that heart rate and activity data (step counts) are not accessible from the wearable device itself and instead intended to be read through applications running on the smartphone (even though the watch is able to collect these data).

To address this limitation, we use external service calls on the linked smartphone (since currently available wearables do not support a direct Internet connection) for collecting these data, as well as for annotating application and notification categories.

**Figure 3:**
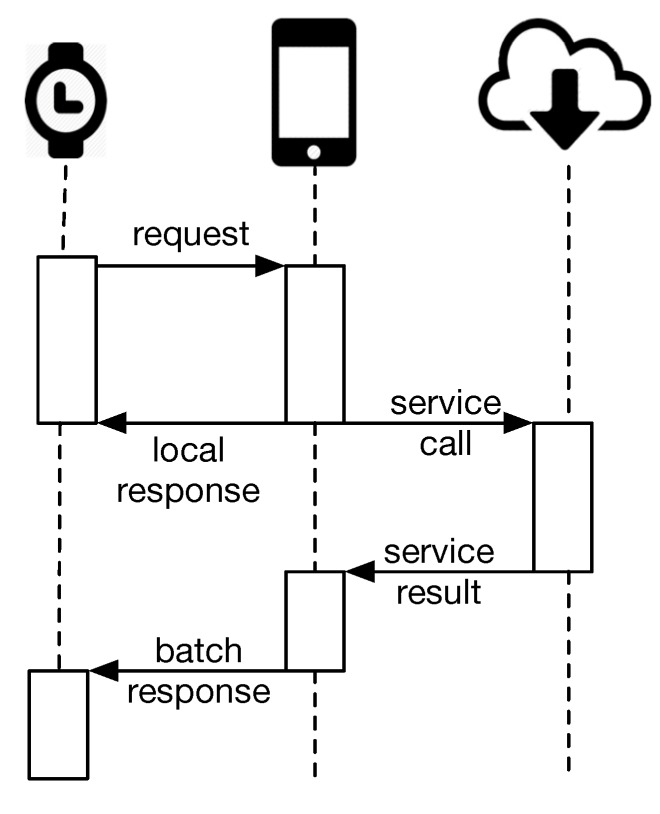
Communicating data between the phone or cloud and the smartwatch. If the data come from the phone, the response is immediately sent to the smartwatch. Otherwise, if there is a need for an external service call via the cloud, the Sensor Reader on the watch performs a batch call without waiting for the response. When the response is ready, it will be delivered to the smartwatch via the phone.

A call to an external web service from the watch is shown in Figure 3. If the data are provided via the phone (local call), such as Google Fit API history, the smartwatch sends a request, and the associated Sensor Reader waits for the data. Otherwise, if the data are via an external service call, the Sensor Reader is not suspended, and when the response is available, a “Batch Response” will be sent to the Sensor Reader, e.g., identifying the category of an application. This approach is necessary to reduce the latency of the application, and in user interfaces, some sensors show “last sync. [date time]”. For example, Figure 6e shows the last synchronization time at which the data were read from the external source.

In the “insight” implementation, we call external services once a day for getting data from the Google Fit API or when the user navigates the associated user interface on the watch. We recommend that developers consider the cost of issuing external calls and be sparing with their use. The cost of transferring data and making external service calls will be examined in Section 5.3.1.

### 4.2. Learning and Prediction

The second part of this framework focuses on learning the user behavior and implementing a novel lightweight prediction based on the users’ contextual data.

As has been stated previously, a prediction or any other reasoning component that runs on small devices must be light enough to handle the limited computing capabilities of these devices. Moreover, we found in one of our experiments (Section 5.3.1) that transferring data from a wearable device is a resource-consuming process. Therefore, a prediction component is useful if it performs the reasoning on the device and thus allows relevant applications to instantaneously benefit from its predictions. Most wearable applications, such as health monitoring, require instant decision making [15]. On the other hand, there are several approaches that benefit from uploaded data and conduct these analysis either offline [17,45] or in the cloud and subsequently stream back the result [16]. Even new approaches for wearable data analysis, which are application specific, are performed on the smartphone [46] and not the wearable device itself. Even though outsourcing data analysis to a remote system is easier from a development perspective, this approach would require a connection to the cloud and causes a significant network overhead on the target application, as well as privacy issues.

In this section, we first discuss data management with prediction. Then, we describe how data will be transformed to an abstraction that assists in the prediction. Following that, we describe our sensor-independent profile creation algorithm and how the implemented app benefits from the profile creation algorithm.

#### 4.2.1. Data Management and Retention

Storage size is not considered a major challenge, but increasing the number of data points for modeling and learning the user profile increases the computational complexity of the prediction algorithm. Our framework therefore operates independently from other devices, and all calculations are done on the smartwatch. Therefore, the complexity of the algorithm has to be designed with limited resources available on the device in mind. In order to resolve this issue, we allow developers to define a data retention policy through our Policy component. By default, we use seven days, which is based on Google’s Fit user interface (on the smartwatch). However, seven days could be too short for the prediction process, especially identifying and predicting weekend behaviors. This could be resolved by weighting the source/data (confidence) in the user profile, which will be described later in this section.

It is noteworthy that keeping or removing the original data is application dependent and our framework does not remove the original data from the storage. However, our prediction suggests keeping at least seven days of data.

We apply a data retention policy to all sensors. It is also possible to define a data retention policy for each sensor, but for the sake of consistency in the user interface, we have not implemented this approach.

#### 4.2.2. Semantic Abstraction

According to Tenenbaum *et al*. [47], generalization from data is a central point of any learning process. In other words, we learn based on generalizing new concepts and then deducing knowledge about the generalized concept.

Raw context sensing data should be able to provide information about the real world. In simple terms, to enable the model providing information about users’ real-world context, raw context sensing data should not be directly fed into a model. For instance, two logs of a user originated from our context sensing components on the smartwatch are as follows:

{"name":"Activity",
 "timestamp":{
  "start_time":"Wed Feb. 25 21:25:01 2015",
   "end_time":"Wed Feb 25 21:38:02 2015"},
 "sensor_data":{"step_count":2438,"delta":29}}
{"name":"Activity",
 "timestamp":{
 "start_time":"Thu Feb 26 21:10:01 2015",
   "end_time":"Thu Feb 26 21:21:23 2015"},
 "sensor_data":{"step_count":1930,"delta":14}}


Humans do not perceive time in and of itself [48], but rather, perceive changes or events in time. To be able to model human behavior, a precise machine timestamp should be transferred to a format similar to the way humans perceive time. Both timestamps in the given examples are not exactly equal. However, based on human perception of time, the difference is not significant; meaning, if we consider the precision of one hour, we can argue that the user walks about 2000 steps daily between 09:00’ p.m. and 10:00’ p.m.

The prediction system should be able to recognize that these records are similar. Therefore, the data need to be converted or normalized to a higher level of abstraction, which enables the system to estimate similarities, similar to human perception. This reveals the need for a semantic abstraction. The concept of semantic abstraction or generalization is not new and has been described, for instance, by Bettini *et al*. [35], who call this concept “situation”. We extend this work by providing a novel semantic abstraction trimmed for smartwatch sensors. It is not necessary to implement semantic abstraction to perform reasoning, and other approaches use raw contextual data for user modeling and prediction [14,16]. However, such a generalization can assist us with converting raw sensor data to semantically-rich, meaningful data (see Section 5.3.3).

The process of transforming data to a higher level knowledge is known as “abstraction” [35], “normalization” [17] and “temporal granularity” [49] for temporal data.

Ma *et al*. [17] provide a higher level abstraction strategy, which converts the applications names to a genre of application, e.g., “Skype” to “Communication”. Rawassizadeh *et al*. [49] argue that human perception of time is different from machine timestamps. They suggest modeling time-based similarity; temporal granularity is required. Temporal granularity is the transformation of a timestamp to a time representation similar to human perception. They demonstrate that temporal granularity of one hour has the highest accuracy in predicting human behavioral motifs. We, therefore use the temporal granularity of one hour in our framework. For example, 11:23’, 11:26’, 11:41’ are similar times, and if we transform them based on “one hour” temporal granularity, we get 11:00’, 11:00’ and 12:00’.

Based on the aforementioned works and existing sensors on smartwatches, we propose to convert context sensing data to a semantic abstraction as described in Table 2. Heart rate discretization has been extracted from existing smartphone heart rate monitor applications in the market. We apply supervised discretization on activity (number of steps) and ambient light based on formative evaluation between developers of the system. The focus of this paper is not to identify the best discretization approach, and this is not discussed in further detail here. The *Semantic Abstraction* component in the architecture is responsible for implementing the process of data conversion.

#### 4.2.3. Profile Creation Algorithm

The first step of the prediction algorithm is to analyze context sensing data for a few days. During the context data collection phase, the system is in the “Cold Start” phase, and we suggest not to run the reasoning component (Alarm and Notification). The duration of the cold start phase depends on the target application usage. For instance, if a target application tries to quantify the user’s physical activities during the week, then at least one week of data is required. In another example, if the target application tries to model and notify about battery discharge routines, two days would be sufficient.

**Table 2:** Semantic abstraction of context sensor data to allow the framework making inferences similar to human perception.

Data SourceName	Original Data	Abstract Data
Timestamp	year/month/day hour:minutes:second	week day/ rounded hour
Activity	run, walk, in vehicle, on bicycle	no change
Activity (step counts)	number of steps	<1000: very inactive 1000–5000: inactive 5000–10,000: active 10,000 >: very active
Heart rate	number	<60 : low 60–90: average >90: high
App usage	app name	app genre
Battery	percentage and charging or discharging status	rounded to 10% change and charging or discharging status
Notification	app name	app genre
Bluetooth	connected or disconnected	no change
Ambient light	number	0: dark 0–10,000: less bright 10,000–50,000: bright >50,000: very bright

**Figure 4:**
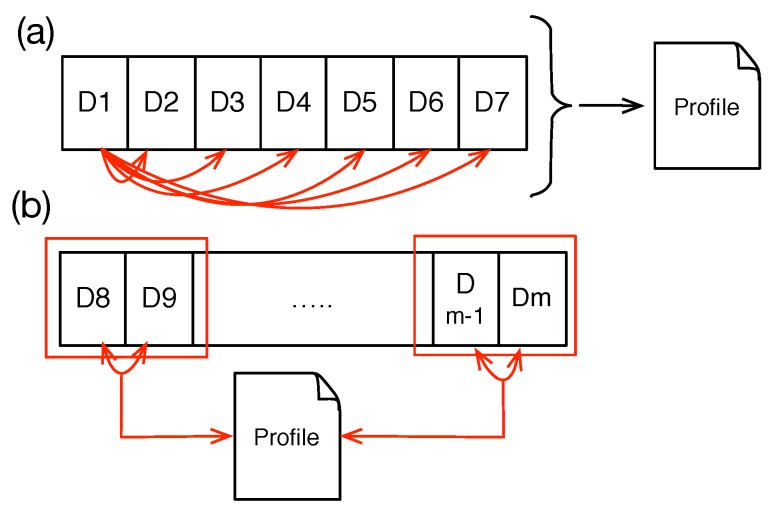
(**a**) Profile creation process in the cold start state. Here, all days will be compared to all other days; (**b**) Profile update after the cold start state, in which two days are compared to each other and then with the profile.

The second step is to compare the similarity between collected contextual data and to build a “profile”, as shown in Figure 4a. In this example, seven days are compared to each other. By “comparison”, we mean comparing records to each other. For *n* days, we will have *n* number of comparisons. Therefore, the computational complexity for the cold start phase is $O(n^{2})$. However, this happens only once in the life time of the system. Even if we consider n=7 (worst case scenario), we will only have 49 comparisons. Moreover, the algorithm does not compare all sensors and only compares the sensor data that have defined an alarm in the policy file. Therefore, as our empirical implementation reveals (Section 5.2.1), the performance impact is too insignificant.

After this, the profile object is created. The profile object is a set of records that contains similar sensor/data, temporal granularity and weekdays of the repeated data. Moreover, a profile object includes confidence, which is the normalized number of repeated data divided by the total days that the target data have been sensed. For instance, if a notification message of a communication app arises six times in ten days between 7:00’ p.m. and 8:00’ p.m., the related record in the profile object will be as follows:

{"confidence":"0.6", "time":"19:00-20:00",
 "app":"communication", "weekdays":["mon","tue","thu","fri"]}


The user profile can also be described as a data dictionary, which includes a summarized version of the user’s frequent behavioral patterns.

After the cold start phase, the algorithm uses the sliding window concept to compare each two days to each other to get their similar records. It then adds them into the profile, or if they exist inside the profile, it increases their confidence, as shown in Figure 4b. It is possible to simply compare each day to the profile object and update the profile object accordingly. However, human interaction and behavior change over time [49]. We therefore did not implement this strategy due to the “concept drift“ caused by the dynamics of human behavior. Concept drift [50] means that properties of the target variable change over time, preventing modeling for prediction. We further use this term to describe behavior that decays over time. In particular, two days will be compared (i) to each other and (ii) to the profile. Moreover, as the number of days increases, the confidence of behaviors, which are not repeated in a profile, decreases. The reasoning component, *Alarm and Notification*, could employ a threshold value to eliminate records with low confidence inside the profile.

**Figure 5:**
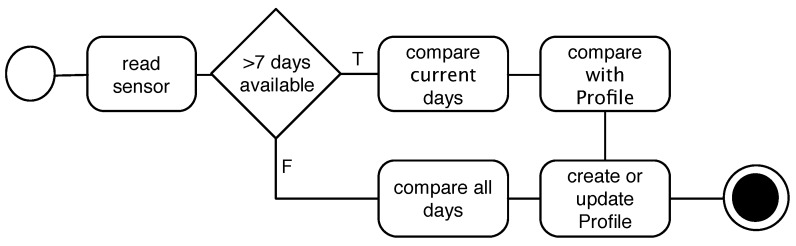
Abstract representation of the profile creation and administration algorithm.

The *Profile Extractor* component in Figure 1 is a representation of our prediction algorithm described above. Figure 5 visualizes our profile creation and administration algorithm.

#### 4.2.4. Alarm and Notification

The Alarm and Notification is a reasoning component, which benefits from the proposed prediction approach. It uses the description inside the policy and has a listener to recognize sensor data changes. If there is a specific sensor that has been mentioned in the Policy file and its data are different from the value inside the profile object, then an alarm will be created and sent to the *Output Interface*. The Output Interface could be a user interface or any communication channel that is used by the app. For instance, it could be an email, and if the target user, who uses an elderly care application, does not have any activity after a specified time, it sends an “email” to the care givers. Following is a simple example of an alarm inside the Policy:

{"alarm":{"sensor":"battery"; "condition":"lower";
"message":"The battery is draining faster than normal."}}


**Figure 6:**
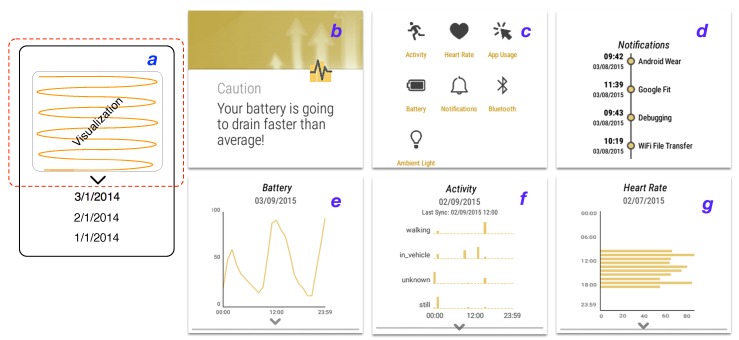
(**a**) The visualization pattern that is used for each sensor. The red area depicts the smartwatch screen. Previous days can be accessed through scrolling; (**b**–**g**) Some of the screens of the implementation of our framework designed for end-user experiments.

The value element inside an alarm can be either “low” or “high”. Low means that now, the target sensor value is lower than the value inside the profile object at the given time. A similar scenario is applicable for the value being set to high. Figure 6a shows a message created based on the alarm set for the battery and sent as a notification message to the smartwatch user interface (UI). Here, the UI is the Output Interface.

## 5. Implementation and Evaluation

This section first describes an application and its user interface, which we developed on top of our architecture. Then, it describes a series of evaluations that demonstrate the utility and efficiency of our integrated context sensing and prediction framework.

We implemented our framework for the Android Wear platform, since at the time, there were several smartwatch brands on the market supporting Android Wear. It further offered an open SDK for development. The implemented prototype was tested with ten participants, aged between 22 and 35, three of them being females. The following brands of smartwatches were used for the user interface implementation: Moto 360, Samsung Galaxy Live, Sony S3, LG G Watch R. We used the Moto 360 and Samsung Galaxy Live devices for other evaluations, but each study reports numbers from one device only.

### 5.1. User Interface Implementation

In order to implement and integrate the proposed framework into an end-user application, we implemented this framework in an application called “insight” on top of our architecture. This application provides users with an overview of their activities, *i.e.*, lifelogging, and device activities through simple visualization for each sensor, similar to other quantified self applications. Dobbins *et al*. [51] provide more detail about the state of the art of lifelogging systems. We designed these visualizations following Starner’s suggestion [52]: *Besides being physically light, consumer wearables must be visually lightweight.* Insight’s main UI, which features a list of senors and sources, is shown in Figure 6c. The visualization for a sensor is activated by “clicking” on the according source icon, as shown in Figure 6.

When the user opens a visualization screen she/he encounters a downside animated caret symbol below each visualization, which notifies the user about the scrolling feature. After the first scroll, the caret symbol fades away. Scrolling the screen enables the user to get information about previous dates, and through clicking on previous dates, the visualization is updated with the data for the selected day. Figure 6 shows the pattern used for different visualizations. Except the timeline-based visualizations, *i.e.*, notification and Bluetooth, only ten days of data are available to scroll and visualize (similar to Google Fit, which displays seven days).

Visualizations are in the form of histograms and combinations of histograms (app usage, heart rate and activity; Figure 6f,g), timelines (notifications (Figure 6d) and Bluetooth connections) and linear plots (battery (Figure 6e) and ambient light).

When the user opens the app, she/he is presented with a screen similar to Figure 6c. Then, by clicking on each sensor, the user can access the related visualization of that sensor. If a sensor requires a service call from the phone, e.g., activity sensor, then the last fetched date-time from the phone will be shown as the “Last Sync” text on its visualization, as shown in Figure 6f.

Due to the application-agnostic approach of our framework, we implemented the prediction for two different sample use cases. The first use case reads the battery sensor and notifies the user about anomalous battery discharge, based on leveraging the history of the battery discharge rate. The second use case notifies the user if she/he is not obliged to the routine of physical activities (activity sensor), based on leveraging the history of their physical activities.

### 5.2. Context Sensing and Data Collection

This section lists and describes evaluations that are related to the contextual sensing module of the framework. As has been described, we introduce a new model for the ambient light context sensing module. Therefore, our first evaluation analyzes the battery utilization differences between our proposed ambient light data collection approach and a baseline. We are using a buffering mechanism to handle parallel writes on the disk. To our knowledge, there is no other study that reports the resource efficiency of using buffer *versus* writing into the smartwatch storage. Therefore, the second evaluation reports the impact of writing into different types of storage with or without a buffer. Afterward, we provide a holistic overview of the framework resource utilization and analyze its battery efficiency in different connectivity (Bluetooth) states. This study demonstrates the overall resource efficiency of our framework.

#### 5.2.1. Ambient Light Data Collection

Balan *et al*. [31] state that ambient light itself is not a resource-consuming sensor. They also report on the impact of writing ambient sensor data into internal flash memory. However, similar to accelerometer, ambient light is very sensitive to small light changes in the context, and thus, logging such a data results in a very large amount of data in the storage. Due to the performance overhead of frequent writing [53], continuous logging of ambient light is not energy efficient. Section 4.1.2 describes our approach to collect ambient light data with two parameters that are application specific. To demonstrate the battery efficiency of our ambient light data collection approach, we compare our approach to a baseline method. The baseline is simply collecting ambient light data when there is a change available and writes the data directly into the SD card storage. In other words, the baseline is not using our proposed approach and simply stores the data as soon as they are available.

**Figure 7:**
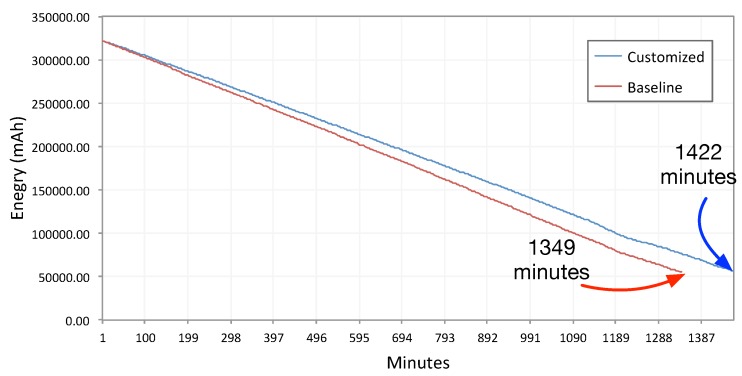
Battery discharge rate comparison between baseline ambient light sensing and our proposed sensing method. The test condition is set to discharge the battery from 100% to 1% .

For this study, we use an SD card as storage. Please note, although there is no slot for SD cards on smartwatches, to stay in line with previous research studies, we call the external storage the SD card. The Android Wear SDK also uses the term ‘SD card’ to refer to the external storage. Figure 7 shows the discharge rate from 100% battery (fully-charged battery) to 1% for both the baseline and our method.

As shown in Figure 7, there is a difference of about 73 min in the battery discharge rate between the baseline and our approach in the 23:42’ duration of the experiment. The battery has been discharged from 100% to 1% in this time.

Figure 7 reports for the Moto 360 device, with 512 MB RAM and 1-GHz single-core Texas Instruments OMAP 3. We set $d_{wakeup}$ to 10 min and $d_{interval}$ to 30 s. This test was conducted in a 4 °C temperature (inside a refrigerator to have a constant temperature); the watch screen was turned off; the device was not moving; and there was no user interaction. Therefore, it was discharged at a slower rate than during normal use. Nevertheless, 73 min in 23:42’ is still a significant difference. Therefore, we cannot conclude that our ambient light sensing approach is resource efficient.

It is notable that, reducing $d_{wakeup}$ and $d_{interval}$ values will bring the discharge rate close to the baseline. However, we do not find any use cases for a data collection similar to the baseline method. Even a listener service, which needs to fetch ambient light frequently and to decide instantly, can use a small size buffer. The impact of buffering on different storage (SD card and internal flash memory) is described in the next section.

#### 5.2.2. Storage and Buffering Impact

Continuously writing context data from different sensors or information sources is a major requirement of the framework. Nevertheless, in contrast to the conventional wisdom, storage affects the performance of the device [53]. Since different information sources (sensors) can write their data in parallel into the storage, it is necessary to queue data before writing. As described above, the Temp Buffer component is responsible for this queuing of data.

Therefore, we analyze whether buffering the data (before writing them onto storage) has an impact on battery utilization.

To evaluate the impact of buffering on the battery performance, we carried out an experiment that involved continuously writing context sensing data onto the SD card and internal flash storage of a smartwatch with different buffer sizes. In other words, this experiment allows us to identify the optimal writing strategy. Since there is a direct correlation between the writing and reading performance [54], we can generalize the findings for writing also to reading. For this reason, we do not analyze the reading operation separately.

**Figure 8:**
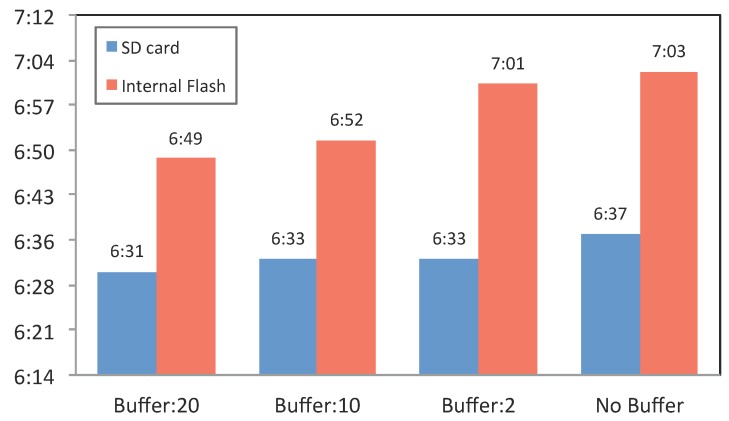
Battery discharge duration from 100% to 10%, with different buffer sizes and different storage locations, *i.e.*, internal flash memory or SD card.

We created a simulator that sent synthetic data, similar to our original contextual data, constantly (each second) to the Sensor Reader component. The Temp Buffer size was set to 2, 10 and 20 records to highlight the impact of different buffer sizes on the battery. Results of the experiment are reported in Figure 8. This study involved eight conditions, which involved disabling or enabling the buffer, combinations of the three different buffer sizes (2, 10 and 20) and storing on internal flash storage or SD card. Figure 8 reports the results for the Samsung Galaxy Live device in a 16 °C temperature.

The results indicate that using the buffer size of 20 results in a very minor increase in battery utilization, especially for the internal storage. For the SD card, this change is even smaller and, therefore, not worth further investigation. The SD card reacts less to the use of buffer size than the internal flash storage. Therefore, we can argue that using the buffering mechanism (utilizing memory) does not affect the performance of the battery while the data are written to the SD card. Although the differences are insignificant, this experiment demonstrates that the memory overhead originating from buffering data has a higher impact on battery performance than writing directly into storage. In simple words, direct writing on the disk is slightly more energy efficient than using memory to cache data and then writing to the disk.

The results further show that writing the data to the SD card consumes more battery than writing to the internal flash storage. This finding is in line with Caroll and Heiser’s analysis [54] (on a smartphone) and Berlin *et al*. [34] (on a wrist-worn activity logger). Both studies identified that writing and reading to/from an SD card consume more power than using internal flash storage. The most notable difference is between writing onto the internal flash storage and SD card with no buffer. Even this difference is only about 25 min, which may not be significant enough to affect the design of a system. The main differences between our study and previous findings is the evaluation with the existing one is the evaluation of buffer usage in order to analyze the buffer impact while writing onto the storage. However, the choice of storage is also application dependent. For instance, if the target application’s goal is long-term large data collection, for example in lifelogging systems [55], the use of an SD card is appropriate due to its larger storage capability. Otherwise, if the data size is not large and needs to be kept temporarily, internal storage is preferred.

For this experiment, we have used a Samsung Galaxy Gear Live, with Qualcomm Snapdragon 400, Quad Core 1.2 GHz Processors and 512 RAM. We kept the screen active with its 50% background light to drain the battery faster.

#### 5.2.3. Overall Framework Impact on Energy

One of the claimed features of the proposed framework is its energy efficiency. Therefore, to demonstrate the overall energy efficiency of our framework, we conducted comparisons of the battery discharge rate across four different states, including Bluetooth on or off and “insight” running or not running. We configured the running state of “insight” as follows: all six sensors were enabled, and the prediction algorithm was executed once every hour. The prediction used ten days of data in each execution.

Table 3 shows the battery discharge rate while running or not running the application, with Bluetooth enabled. There is only about an 11-min difference in about 23 h, when switching the smartwatch to airplane mode. Moreover, there is only about a 31-min difference in about 20 h, when the phone is connected to the watch. These results demonstrate that the discharge rate is insignificant while running the implementation of our framework. However, since notifications from the phone were streamed into the device continuously, the gap is a bit larger in comparison to the same settings in airplane mode. Nevertheless, the differences can be considered insignificant, and the results show that the implementation of our framework on the smartwatch is indeed resource efficient.

**Table 3:** Overall battery consumption of insight while running on the watch.

Bluetooth Status	Insight Status	Discharge Duration
on	on	19:43’ (1183 min)
on	off	20:14’ (1224 min)
off (airplane mode)	on	23:08’ (1388 min)
off (airplane mode)	off	23:19’ (1399 min)

To implement this test, we monitored the discharge duration from 100% until the battery reached 5%. To prevent bias originating from the device state, we kept the device settings and user activities the same in all four test conditions. Table 3 reports the results for the Moto 360 device. The experiment was conduced in a 4 °C temperature. The device was not in use by the test administrator (kept inside the refrigerator). However, notifications were streamed from the phone to the device, when they were connected. Therefore, we can argue that the conditions across tests were constant and equal.

We do not report on each sensor individually, as they collect data via the operating system level API, and therefore, no connection to Bluetooth is required. In other words, the resource efficiency impact of software API-based sensors is too insignificant and not worth any further investigation.

At the time of writing this paper, most existing Android-based smartwatches support only Bluetooth and no other radio frequency-based sensors. Therefore, after the screen, which is the main battery challenge, the Bluetooth connectivity is the second challenge, as it requires radio-based communication.

### 5.3. Prediction and Information Retrieval

As described earlier, the integration of the prediction process into the device in parallel with context sensing is one of the major novelties of the proposed framework. This section first provides a comparison between the cost of on device analysis and transferring data into another medium, *i.e.*, smartphone or cloud. The second evaluation analyzes a file structure to identify the fastest data access approach on the device, which is used by data analytic approaches (prediction). Semantic abstraction is another feature of the framework that we have proposed, and the third study focuses on evaluating the impact of the semantic abstraction of prediction accuracy with well-known predictive analytic methods.

#### 5.3.1. Data Transfer Cost

As described earlier, there is a lack of light and generic prediction mechanism that can operate on small devices, such as smartwatches. The proposed framework fills this gap. Transferring the data to the smartphone increases the resource costs, and also, transferring from the smartphone to the cloud incurs an additional cost, disregarding the privacy risks associated with sharing context sensing data [12].

Our prediction method is light enough to run on the device. Therefore, we claim that running the prediction on the device significantly reduces the cost of preparing the data objects and transferring them via Bluetooth to the smartphone. In particular, the minimum cost includes (i) compressing files and (ii) streaming them through the Bluetooth to the smartphone, and then, the smartphone should (iii) read the stream, (iv) decompress the files and (v) run the prediction. If we hand over the prediction process to the cloud, the smartphone has the cost of transferring the data to the cloud, but removes the cost of running the prediction on the smartphone. In order to demonstrate the resource efficiency of our approach (that performs the prediction on the device), we propose a baseline method that collects the data, compresses them (to reduce network bandwidth) and sends them to the phone. Then, the data analysis could be done on the phone. Although there is additional cost of data analysis on the phone or in the cloud, we do not add that cost to the baseline. In other words, even the baseline neglects the cost of (iii) and (iv) and only considers (i), (ii) and (v) as the cost.

A comparison between the baseline method and our method could demonstrate the resource efficiency of our approach.

**Figure 9:**
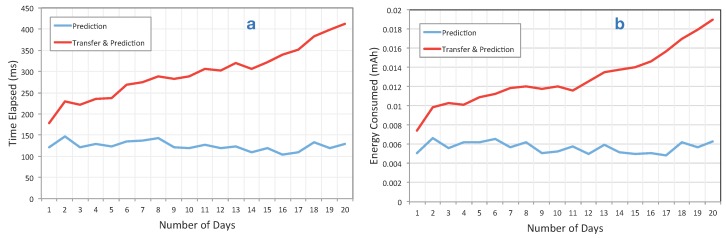
(**a**) The execution time differences while running the algorithm on the device (blue) *versus* transferring it to the phone and running it there, *i.e.*, baseline (red); (**b**) the battery consumption in mAh between the baseline *versus* our on device prediction approach. As shown, our method consumes less battery, and its execution time is significantly lower than the baseline. This difference will be highlighted when the number of days increases.

Figure 9 shows the differences between our approach and the baseline. As described, we implemented the prediction for two different sensors, *i.e.*, battery and physical activities. The prediction for both use cases ran each two hours; for instance, in 10 h, the prediction was executed five times. In the test scenario, we used 20 days, which include about 11,246 records. Logs file sizes for each days were not equal, but on average, we can say about 1100 records each day.

As illustrated in this figure, our approach consumes significantly less battery compared to the baseline. Moreover, the execution time of running the prediction on the device is significantly shorter than running it on the phone. Since the cost of transfer is more than running the prediction, it is clear that running it on the cloud is even less resource efficient than running it in the phone.

The cost difference is highlighted especially as the number of data records/days increases. As shown in Figure 9, the performance of our prediction algorithm does not changes, even if the number of days increases. This is useful in several applications, e.g., quantified self applications, which rely on historical data, and data analysis in those applications requires accessing the a history of data.

#### 5.3.2. File Structures and Search

The problem of searching and information retrieval in contextual data has not yet been widely explored in mobile sensing and other contextual data collection paradigms. Continuous context sensing applications can collect large amounts of data, and searching them on the device is a resource-intensive process. For instance, if an application collects GPS and WiFi data and the user is driving for many hours within a city, there will be many WiFi and GPS instances in the dataset. Therefore, subtle approaches are required for reducing the search space.

Furthermore, search is necessary for some context sensing-based applications, such as life logs. Existing commercial life log applications, such as Sony’s Lifelog (http://www.sonymobile.com/us/apps-services/lifelog), provide browsing, but they do not support search. Although the focus of this paper is not to tackle the search issue, we suggest that the continuous context sensing process should provide mechanisms for facilitating efficient context information retrieval.

Additionally, contextual information sources are heterogeneous, and traditional relational database systems are not optimized for information access. For instance, one can save Bluetooth BSSID, timestamp of connection status change and device name of the Bluetooth sensor (four data objects) or only the application name and timestamp for the application sensor (two data objects).

Our Context Storage component uses a file structure to store data and the JSON format to handle the heterogeneity of data. The flat file structure for data storage and JSON format to handle heterogeneity were used in similar context sensing approaches for smartphones, such as UbiqLog [19]. To gain a deeper overview of the best storage approaches, we propose and compare three different strategies for storing context data: (i) all source data are being stored in a single file, and a file is being created per day (baseline); this approach has been used in previously mentioned works; (ii) for each source, a separate folder is being created, and for each day, a new file will be created inside the target folder, *i.e.*, source-folder-day (SFD); this file only contains one type of source data; (iii) for each day, a new folder is being created, and all sources’ data will be recorded in a file inside that folder, *i.e.*, day-folder-source (DFS).

**Figure 10:**
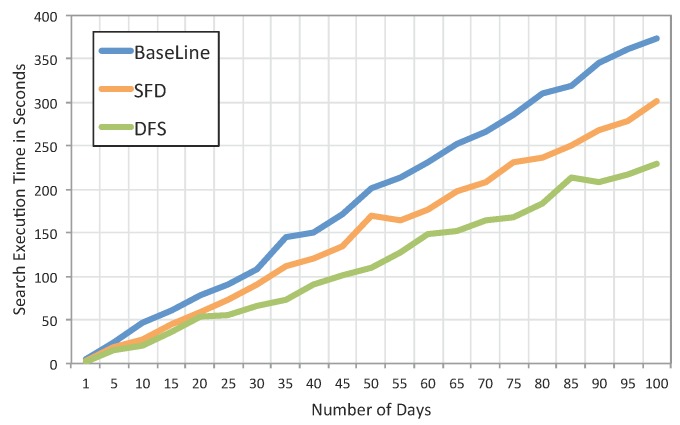
Search execution time for three different proposed data structures.

We then compared the search time through traversing these file structures for one randomly-chosen source (*i.e.*, Notificationsource). In particular, the test uses a cursor that opens each file and reads the file content line by line.

Figure 10 shows differences between the three strategies. Separating sensors yields better performance rates, as this strategy reduces the search space. Therefore, SFD and DFS both outperform the baseline strategy. As shown in Figure 10, increasing the number of days increases the search execution time. For instance, for 100 days of data, which is about only three months of data, the experiments show 144-s differences between the baseline and SFD. This means that SFD is the most efficient file structure for data storage in our framework.

#### 5.3.3. Semantic Abstraction and Accuracy of Prediction

As described earlier, our proposed system includes a novel approach to semantic abstraction. It facilitates reasoning approaches, such as prediction, through generalizing and discretizing the data. The prediction algorithm we described relies on semantic abstraction. For instance, a user’s battery shows a level of 65% at 11:05’ on the first day, 67% at 11:15’ on the next day and 40% at 11:20’ on the third day. If the framework does not use a generalization approach, the Alarm and Notification component cannot distinguish differences between the first two days and the third day. This means the battery levels on the first day and the second day are similar; thus, the Profile Creation algorithm (prediction) brings them to the profile object. Semantic abstraction allows this component to identify that the first two days are similar, and on the third day, an alarm, such as shown in Figure 6a, is raised to alert the user about the unusual drainage pattern of his/her battery.

In order to demonstrate the impact of semantic abstraction on the prediction algorithm, we use 10 days of real data collected from nine participants who volunteered to share their data with one of the authors of this paper. They accept to share their data to be used for this specific analysis with the specified authors who analyze the data. Before participation in the study, they were briefed about the privacy implications that they might face as a result of sharing their data. In all instances, we sought their agreement through the administration and signing of ethics consent forms. Moreover, they were able to disable or enable the “insight” manually or to withdraw from the study at any time. In general, the dataset contains the following data: 4538 activity logs from Google Play API (in vehicle, running or walking duration), 12,963 walking and running step counts, 286 Bluetooth connections or disconnections records, 95 heart rate records, 13,050 ambient light changes and 10,087 notifications that were streamed to the watch. As described earlier, all data were timestamped. We calculated the prediction for each participants separately, which was then aggregated in the form of averages and reported here.

We propose three different settings to compare the accuracy of prediction and to demonstrate the impact of semantic abstraction on predictability. The first setting, which serves as a baseline, uses raw data with no semantic abstraction and real timestamps (no temporal granularity). The second setting again uses raw data, but it applies temporal granularity on the timestamp and converts them based on temporal granularity (TG) of one hour. The third setting applies both temporal granularity on timestamp and semantic abstraction (TG-SA).

Applying a generalization, the algorithm reduces the accuracy of the data, but improves the predictability. Semantic abstraction is a generalization and discretization applied on source data. Therefore, our initial hypothesis assumes that the TG-SA approach has the highest accuracy of predicting the “original data” and the baseline has the lowest accuracy of predicting the same data.

To evaluate this hypothesis, we used four well-known classifier algorithms; naive Bayes, SVM, decision tree (C4.5) and logistic regression. For the implementation of these classifiers, we used Weka v.3-7 (www.cs.waikato.ac.nz/ml/weka) without changing the default parameters for each algorithm. For instance, the polynomial kernel was used for the SVM algorithm. Banaee *et al*. [56] provide detailed information about the challenges of data mining algorithms for wearable data.

For each classification, we used 10-fold cross-validations. In short, 10-fold cross-validation breaks the data into 10 sets (total size divided by 10) and trains nine datasets and tests them on the remaining one. It repeats this process 10 times and reports a mean accuracy at the end. Table 4 shows the receiver operating characteristic (ROC) area, precision, recall and f-measure for each algorithm and the three aforementioned settings. As shown in Table 4, TG-SA has the highest predictability in all test algorithms followed by TG and then the baseline. This result confirms our initial hypothesis. The accuracy of prediction depends on the algorithm used, but all of the algorithms show improvement on the prediction accuracy through using semantic abstraction and temporal granularity.

**Table 4:** Prediction accuracy improvement, by applying temporal granularity (TG) on timestamp and semantic abstraction (SA) on sensor data.

Classification Algorithm		BaseLine	TG	TG-SA
Naive Bayes	ROC-area	0.82	0.93	0.94
	Precision	0.56	0.65	0.66
	Recall	0.69	0.73	0.74
	F-Measure	0.58	0.65	0.68
SVM	ROC-area	0.79	0.86	0.86
	Precision	0.70	0.69	0.72
	Recall	0.74	0.75	0.78
	F-Measure	0.67	0.73	0.75
Decision Tree	ROC-area	0.72	0.70	0.72
	Precision	0.46	0.46	0.55
	Recall	0.65	0.64	0.68
	F-Measure	0.54	0.54	0.57
Logistic Regression	ROC-area	0.79	0.83	0.89
	Precision	0.66	0.69	0.64
	Recall	0.71	0.75	0.78
	F-Measure	0.64	0.65	0.68

The decision tree algorithm has the lowest ROC-area in comparison to the other algorithms. This is due to the fact that the decision tree itself can be used for discretization of continuous data into discrete data, and we have not fed the model any continuous data. The decision tree does have lower performance with discrete data in comparison to other well-known algorithms, such as naive Bayes [57]. We discretized all data to enable a similar calculation for the profile creation algorithm.

Our profile creation algorithm operates on semantic abstraction data and temporal granularity of one hour’s time. Due to the cold start situation, uncertainty originating from wearable and mobile data and resource limitations, we do not recommend using these well-known algorithms for a prediction component that runs on small devices, such as smartwatches.

It is important to point out that this section does not focus on evaluating the predictability of the collected information. Instead, it demonstrates the utility of semantic abstraction and temporal granularity on increasing the accuracy of prediction.

## 6. Conclusions and Future Work

In this paper, we have described a novel, lightweight, energy-efficient framework that integrates continuous context sensing with prediction for wearables, such as smartwatches. Our implementation of the framework “insight” supports existing sensors on Android smartwatches. The architecture of our framework is hardware independent and uses a flexible approach to host new types of sensors. Moreover, this framework contains a light prediction component that can operate on a short cold start phase (two days at minimum) and runs on the device. This prediction system benefits from a novel, semantic abstraction that discretizes and generalizes raw data to a higher level abstraction, which facilitates reasoning.

We evaluated the proposed approach through six different experiments. Four experiments were focused on battery efficiency, where we analyzed the ambient light sensing, overall framework and prediction energy efficiency. Moreover, from the energy consumption perspective, we found that directly writing into storage is not much different than caching data in memory from the battery usage perspective. We demonstrated the reduction in search execution time through using the optimal file storage. We analyzed the impact of semantic abstraction on the predictability of the data to demonstrate the effectiveness of semantic abstraction for increasing the accuracy of prediction.

As future work, we plan to evaluate “insight” through a large-scale deployment and to collect data in a privacy-preserving manner. This will allow us to analyze the correlations between data collected from different sensors.
